# Adherent/invasive *Escherichia coli* (AIEC) isolates from asymptomatic people: new *E. coli* ST131 O25:H4/H30-Rx virotypes

**DOI:** 10.1186/s12941-018-0295-4

**Published:** 2018-12-10

**Authors:** Edwin Barrios-Villa, Gerardo Cortés-Cortés, Patricia Lozano-Zaraín, Margarita María de la Paz Arenas-Hernández, Claudia Fabiola Martínez de la Peña, Ygnacio Martínez-Laguna, Carmen Torres, Rosa del Carmen Rocha-Gracia

**Affiliations:** 1 Benemérita Universidad Autónoma de Puebla, Posgrado en Ciencias Microbiológicas, Centro de Investigaciones en Ciencias Microbiológicas, Instituto de Ciencias, Puebla, Mexico; 2 Área de Bioquímica y Biología Molecular, Universidad de La Rioja, Logroño, Spain

**Keywords:** AIEC, IBD, Crohn’s disease, Virotype

## Abstract

**Background:**

The widespread *Escherichia coli* clone ST131 implicated in multidrug-resistant infections has been recently reported, the majority belonging to O25:H4 serotype and classified into five main virotypes in accordance with the virulence genes carried.

**Methods:**

Pathogenicity Islands I and II (PAI-I and PAI-II) were determined using conventional PCR protocols from a set of four *E. coli* CTX^R^ ST131 O25:H4/H30-Rx strains collected from healthy donors’ stool. The virulence genes patterns were also analyzed and compared them with the virotypes reported previously; then adherence, invasion, macrophage survival and biofilm formation assays were evaluated and AIEC pathotype genetic determinants were investigated.

**Findings:**

Non-reported virulence patterns were found in our isolates, two of them carried *satA*, *papA*, *papGII* genes and the two-remaining isolates carried *cnfI*, *iroN*, *satA*, *papA*, *papGII* genes, and none of them belonged to classical ST131 virotypes, suggesting an endemic distribution of virulence genes and two new virotypes. The presence of PAI-I and PAI-II of Uropathogenic *E. coli* was determined in three of the four strains, furthermore adherence and invasion assays demonstrated higher degrees of attachment/invasion compared with the control strains. We also amplified *intI*1, *insA* and *insB* genes in all four samples.

**Interpretation:**

The results indicate that these strains own non-reported virotypes suggesting endemic distribution of virulence genes, our four strains also belong to an AIEC pathotype, being this the first report of AIEC in México and the association of AIEC with healthy donors.

## Background

*Escherichia coli* is one of the predominant Gram negative bacterial species of the intestinal microbiota. It mainly colonizes the gastrointestinal tract but also extra intestinal environments. Among *E. coli* strains there are some considered pathogens and others pathobionts, this depending on the virulence factors that they expressed. *E. coli* genetic variability is caused mostly by horizontal gene transfer, acquiring virulence factors and antibiotic resistance genes. This acquisition is mediated by mobile genetic elements (MGEs), such as transposons, plasmids, bacteriophages and Pathogenicity Islands (PAI) [1]. These elements enhance bacterial capacity to survive in the host environment and to adapt to it. A well accepted infectious disease paradigm indicates that the development of antibiotic resistance allows susceptible species to overtake resistant species [1]. Nevertheless, the increasing evidence of the rising threat of antibiotic resistant bacteria suggests that the evolution of resistance may be more associated with a fitness advantage, including enhanced virulence [2, 3].

Extended-spectrum β-lactamases (ESBL) are enzymes that hydrolyze penicillins by disruption of β-lactam ring and also third generation cephalosporins [4]. The largest group of ESBL are the cefotaximases (CTX-Ms), which have become globally disseminated, being *bla*_*CTX*-*M*-*15*_ and *bla*_*CTX*-*M*-*14*_ the predominant genotypes. This group of ESBL restricts treatment options, increasing the use of carbapenems, and leading to the emergence and spread of carbapenemase-producing *Enterobacteriaceae* [5, 6]. There is an increasing prevalence of β-lactamase resistance due to ESBL, particularly the presence of CTX-M enzymes, and associated fluoroquinolone resistance in MGEs in Extra-Intestinal Pathogenic *Escherichia coli* (ExPEC), being a serious global clinical problem during the last decade [7].

The Type I fimbrial adhesin FimH has been associated with *E. coli* pathogenicity because some *fimH* variants enhance uroepithelial colonization [8]. Furthermore, the *fimH*-30 variant has been linked with high fluoroquinolone resistance levels simultaneously with ESBL CTX-M-15 production (H30-Rx) [9, 10]. Additionally, *E. coli* isolates can be classified using the multilocus sequence typing (MLST) technique, sequencing seven housekeeping genes (*adk*, *fumC*, *gyrB*, *icd*, *mdh*, *purA*, *recA*). Using the MLST scheme, ST131 clones have been classified, which have been identified worldwide spread [11, 12]. Increasing prevalence of antibiotic resistance and ESBL CTX-M-15 production in UPEC strains has been linked to this sequence type [13–18].

Clermont et al. [19], established a method based on multiplex PCR for *chuA*, *yjaA*, TspE4.C2, *arpA* and *trpA* genes, classifying *E. coli* strains into seven phylogroups and one clade. *E. coli* ST131 strains belong to phylogenetic group B2 in subgroup I, [19, 20] and they belong mostly to the O25:H4 serotype, although some strains have been found to be O16:H5 serotype [21–27]. It is well known that B2 strains harbor several virulence factors and there is a scheme that classifies the *E. coli* ST131 into five virotypes (A to E). These virotypes depend on the presence or absence of *pap* (adhesin-encoding P fimbriae), *cnfI* (cytotoxic necrotizing factor), *sat* (secreted autotransporter toxin), *kpsMII* (group 2 capsule synthesis), *iroN* (catecholate siderophore receptor), *afa/draBC* (Afa/Dr adhesins), *ibeA* (invasion of brain endothelium), *hlyA* (alpha-hemolysin) and *cdtB* (cytolethal distending toxin) genes (Table 3). This scheme has been useful to infer virulence in strains isolated worldwide and to determine intercontinental spread [11, 14, 16, 28, 29]. ST131 strains have been linked with community- and hospital-acquired urinary tract infections (cystitis and pyelonephritis) worldwide, but also have been reported to cause other infections as bacteremia, intra-abdominal and soft tissue infections, meningitis, epididymo-orchitis, osteoarticular infections, myositis and septic shock [30–38].

There are six well characterized Intestinal or Diarrheagenic *E. coli* pathotypes: Enterohemorrhagic *E. coli* (EHEC), enteroaggregative *E. coli* (EAEC), enterotoxigenic *E. coli* (ETEC), enteropathogenic *E. coli* (EPEC), enteroinvasive *E. coli* (EIEC) and diffusely adherent *E. coli* (DAEC), all of them displaying a broad range of virulence factors affecting critical host cell processes [39, 40]. The *E. coli* strains that cause extra intestinal infections are currently known as ExPEC, and they are the etiological agent of 80% of urinary tract infections (UTIs) [39]. They are also a frequent cause of peritonitis and neonatal meningitis [41]. In addition to these *E. coli* pathogenic groups, a new pathotype of adherent/invasive *E. coli* (AIEC) was recently described and characterized, and it has been involved in inflammatory bowel diseases such as Crohn’s disease and ulcerative colitis [42, 43].

AIEC adheres and invades epithelial cells and replicates into macrophages [42, 44]. Its adhesion is mediated by binding of the type 1 pili to the host glycoprotein carcinoembryonic antigen-related cell adhesion molecule 6 (CEACAM6) on the intestinal epithelial cells [45, 46]. The type 1 pilus is present in almost all *E. coli* strains and is known to bind mannose sugar receptor sequences found on host cell surfaces [47]. There are two well characterized prototype AIEC strains, LF82 and NRG857c [42, 43]. Both strains are phylogenetically related to ExPEC, they belong to serotype O83:H1, to the B2 phylogenetic group and they have been related with the presence of *insA*, *insB* (which encodes a transposase in *IS*1) and *vat* (which encodes for a vacuolating autotransporter toxin) genes [48, 49].

The aims of this study were to determinate the virulence patterns and the pathotype on a collection of MDR *E. coli* ST131 O25:H4/H30-Rx strains recovered from asymptomatic donors.

## Methods

### Bacterial strains and culture conditions

A collection of four *E. coli* CTX^R^ ST131 O25:H4/H30-Rx strains, isolated from healthy donors’ stool samples and belonging to the phylogenetic group B2 (C7223, C7225, C7226 and C7230) which had specific resistance patterns and its *fimH* variant determined, were used in this study (Table 1) [50]. Additionally, *E. coli* C600, O157-H7 (EHEC), B171-8 (EPEC), EAEC, ETEC and CFT073 (UPEC) strains were used as controls. All strains were grown at 37 °C in LB (Luria–Bertani) media. For adherence and invasion assays, after the overnight culture, *E. coli* B171-8 strain was incubated in DMEM (Dulbecco’s Modified Eagle’s Medium) at 37 °C.

**Table 1:** Characteristics *E. coli* CTX^R^ ST131 O25:H4/H30-Rx strains. Data obtained from Cortés-Cortés et al. [50]

Strain	β-lactamic Resistance profile	Non-β-lactamic Resistance profile	Genetic resistance determinant	Phylogenetic group	ST/ST complex	AIEC genes^a^	Virulence genes^a^
C7223	AMP, AMC, CTX, CAZ	NA, CIP, S, T, TE, SXT	*bla*_*CTX*-*M15*_, *bla*_*OXA*-*1*_, *aac(6′)*-*Ib*-*cr*, *tet(A)*	B2	4225/131	*insA*, *insB*, *intI*1	*fimH*, *iha*, *iucD*, *satA*, *papA*, *papGII*
C7225	AMP, AMC, CTX, CAZ, FEP	NA, CIP, GM, AK, T, TE,	*bla*_*CTX*-*M15*_, *bla*_*OXA*-*1*_, *aac(6′)*-*Ib*-*cr*, *tet(A)*, *aac(3′)*-*II*	B2	131/131	*insA*, *insB*, *intI*1	*fimH*, *iha*, *iucD*, *satA*, *papA*, *papGII*
C7226	AMP, AMC, CTX, CAZ, FEP	NA, CIP, GM, AK, T, TE	*bla*_*CTX*-*M15*_, *bla*_*OXA*-*1*_, *aac(6′)*-*Ib*-*cr*, *aac(3′)*-*II*	B2	131/131	*insA*, *insB*, *intI*1	*fimH*, *iha*, *iucD*, *cnfI*, *iroN*, *papA*, *papGII*
C7230	AMP, CTX, CAZ, FEP, IMP	NA, CIP, S, GM, T, TE	*bla*_*CTX*-*M15*_, *tet(A),*	B2	131/131	*insA*, *insB*, *intI*1	*fimH*, *iha*, *iucD*, *cnfI*, *iroN*, *papA*, *papGII*

*CTX*^*R*^ Resistant to cefotaxime, *O25:H4/H30-Rx* serotype/*fim*H variant-resistance to fluoroquinolones simultaneously with ESBL CTX-M-15 production, *AMP* ampicillin, *AMC* amoxicillin/clavulanic acid, *CTX* cefotaxime, *CAZ* ceftazidime, *FEP* cefepime, *IMP* imipenem, *NA* nalidixic acid, *CIP* ciprofloxacin, *AK* amikacin, *GM* gentamicin, *T* tobramicin, *TE* tetracycline, *SXT* sulfamethoxazole trimetoprim, *S* streptomycin

^a^This study

### Strains characterization by the presence of virulence and AIEC related genes

Specific primers were used for amplification of PAI-I_CFT073_ (RPAi and RPAf) and PAI-II_CFT073_ (Cft073.2Ent1 and cft073.2Ent2), PAI-I_J96_ (*papG*If and *papG*Ir) and PAI-II_J96_ (*hlyD* and *cnf*), *iucD*, *satA*, *papA*, *papGII*, *papGIII*, *cnfI*, *iroN*, *afa*, *afa/draBC*, *ibeA*, *hlyA*, *cdtB*, *neuC*-KI, *intI*1, *insA*, *insB*, *vat*, *bfpA*, *stxI*, *stxII*, thermo-labile toxin, thermo-stable toxin, EAEC plasmid, *eae*, *eaf* and *daaE* genes for multiplex and simplex conventional PCR protocols (Table 2).

**Table 2:** Specific primers used in this study

Target	Primer name	Sequence 5′ to 3′	Tm ( °C)	Amplicon size	Reference
PAI-I_CFT073_	RPAi	GGACATCCTGTTACAGCGCGCA	65	925 bp	[68]
	RPAf	TCGCCACCAATCACAGCGAAC			
PAI-II_CFT073_	Cft073.2Ent1	ATGGATGTTGTATCGCGC	55	400 bp	[69]
	cft073.2Ent2	ACGAGCATGTGGATCTGC			
PAI-I_J96_	PapGIf	TCGTGCTCAGGTCCGGAATTT	57.7	400 bp	[68]
	PapGIr	TGGCATCCCACATTATCG			
PAI-II_J96_	Hlyd	GGATCCATGAAAACATGGTTAATGGG	59.3	2.3 kb	[70]
	Cnf	GATATTTTTGTTGCCATTGGTTACC			
*fimH*	FimH-F	CACTCAGGGAACCATTCAGGCA	57	975 bp	[71]
	FimH-R	CTTATTGATAAACAAAAGTCAC			
*Iron*	IRON-F	AAGTCAAAGCAGGGGTTGCCCG	60	667 bp	[16]
	IRON-R	GACGCCGACATTAAGACGCAG			
*afa* operon	AFA025-F	GAGTCACGGCAGTCGCGGCGG	55	207 bp	[72]
	AFA025-R	TTCACCGGCGACCAGCCATCTCC			
*afa/draBC*	Afa-DraF	GGCAGAGGGCCGGCAACAGGC	60	559 bp	[68]
	Afa-DraR	CCCGTAACGCGCCAGCATCTC			
*ibeA*	IBEA 10 F	AGGCAGGTGTGCGCCGCGTAC	60	170 bp	[68]
	IBEA 10 R	TGGTGCTCCGGCAAACCATGC			
*cnfI*	cnf-f	ATCTTATACTGGATGGGATCATCTTGG	60	974 bp	[73]
	cnf-r	GCAGAACGACGTTCTTCATAAGTAT			
*cdtB*	cdtB-f	AACTGATTTTCGCGTTGCGA	60	741 bp	This study
	cdtB-r	GATACGCCAACAGGGAAATG			
*neuC*-*KI*	kpsII-f	GATACGCCAACAGGGAAATG	63	272 bp	[68]
	kpsII-r	CATCCAGACGATAAGCATGAGCA			
	KI-f	TAGCAAACGTTCTATTGGTGC		153 bp	
*insA*	insA-f	GGCATCCAACGCCATTCAT	62	178 bp	This study
	insA-r	TGTCCCTCCTGTTCAGCTACTGA			
*insB*	insB-f	ATGTTCAGATAATGCCCGATG	62	461 bp	This study
	insB-r	CGTTGGCCTCAACACGATTT			
*vatA*	vatA1076F	CCTGGGACATAATGGTCAGAT	61	330 bp	Arenas-Hernández unpublished data
	vatA1406R	CTGGCAATATTCACGCTACTG			
*vatP*	vatP-86F	TAGCGCGCAATTCAACAATA	61	226 bp	Arenas-Hernández unpublished data
	vatP226R	GCAGATAGTGCCAGAGAGGTAAG			
*intI1*	IntI1-F	GGGTCAAGGATCTGGATTTCG	62	483 bp	[74]
	IntI1-R	CGACGATGATTTACACGCATGT			
*papa*	papA-45F	CAGATATCTCGGTGTGTTCAGTAA	61	641 bp	Arenas-Hernández unpublished data
	papA + 31R	GGTCTTGCCTCACCCTGTAA			
*iha*	ihaEMSAR	CGGAATTCCGATCTCCGATCATGTTAACCG	61	150 bp	[75]
	ihaEMSAL	CGGAATTCCGGCATGCCGAGGCAGTCGTTA			
*iucD*	iucD-30F	GCTGTGGCTGGTAACTCAGG	58	512 bp	Arenas-Hernández unpublished data
	iucD512R	TGCTTCACACAGGGTGGTAAAT			
*fliC*	FliC 242F	GCTGTCCGAAATCAACAACAA	58	304 bp	Arenas-Hernández unpublished data
	FliC 445 R	GGCTATCGTACCGGAACCATT			
Fimbrial adhesin subunit	*daaE*-F	TGACTGTGACCGAAGAGTGC	48	380 bp	[76]
	*daaE*-R	TTAGTTCGTCCAGTAACCCCC			
IS3 Transposase family	STI-F	TTAATAGCACCCGGTACAAGCAGG	64	147 bp	[77]
	STI-R	CTTGACTTCTTCAAAAGAGAAAATTAC			
Heat-stable enterotoxin	STaII-F	TTGTCTTTTTCACCTTTCCC	60	93 bp	[78]
	STaII-R	ACAAGCAGGATTACAACACA			
Heat-labile enterotoxin	LT-F	GGCGACAGATTATACCGTGC	60	750 bp	[79]
	LT-R	CCGAATTCTGTTATAATATGTC			
Intimin	*eae*-F	CAGGTCGTCGTGTCTGCTAAA	67	1087 bp	[80]
	*eae*-R	TCAGCGTGGTTGGATCAACCT			
Shiga toxin 1	STx1-F	TTTACGATAGACTTCTCGAC	55	227 bp	[81]
	STx2-R	CACATATAAATTATTTCGCTC			
Shiga toxin 2	STx2-F	CCCAGTCACGACGTTGTA	60	460 bp	[78]
	STx2-R	TATACTATCGTGCCTTTCCA			
*ial*	ial-F	CTGGATGGTATGGTGAGG	60	320 bp	[82]
	ial-R	GGAGGCCAACAATTATTTCC			
*EAF*	EAF-F	CAGGGTAAAAGAAAGATGATAA	58	1087 bp	[83]
	EAF-R	TATGGGGACCATGTATTATCA			
*bfpA*	*bfpA*-F	AATGGTGCTTGCGCTTGCTGC	67	326 bp	[79]
	*bfpA*-R	GCCGCTTTATCCAACCTGGTA			
EAEC plasmid	EAEC-F	CTGGCGAAAGACTGTATCAT	60	630 bp	[84]
	EAEC-R	CAATGTATAGAAATCGCTGTT			

### Adherence assay

HeLa cells were seeded on tissue culture plates in Minimum Essential Media (MEM) (Thermo Fisher Scientific) supplemented with 10% fetal bovine serum (FBS) (Thermo Fisher Scientific) at 37 °C in 5% CO_2_ until sub-confluence. Then, 5 mL of FC Wash solution with 0.25% trypsin solution was added, incubated 3 min at 37 °C and decanted. Fresh MEM + 10%FBS was added. Cells were adjusted to 5 × 10^4^/mL, 425 µL were seeded on each well of an eight-well Millicel^®^ EZ slides (Merck Millipore). The slide was then incubated overnight at 37 °C in 5% CO_2_. HeLa cells monolayers were washed with sterile PBS. After washing, 250 µL of bacterial suspension in MEM supplemented with 1% mannose were added in each well (1:20) and incubated for 2 h at 37 °C in 5% CO_2_. After incubation, wells were washed twice with PBS. Methanol was used to fix cells monolayers for 10 min and samples were stained with Giemsa. The adhered bacteria number was directly counted microscopically in at least 14 fields of each well; result is expressed as the average bacteria number per cell [42].

### Invasion assay

For invasion assays HeLa cells were grown until 70% to 80% confluence and used to seed 8 well glass slides (Millicel ^®^ EZ slides) with a concentration of 5 × 10^4^ mL and incubated overnight at 37 °C in 5% CO_2_. Monolayers were washed with sterile PBS. After washing, the slides were inoculated with a suspension (1:20) of bacteria in MEM supplemented with 1% mannose. Slides were incubated 3 h at 37 °C in 5% CO_2_ and washed with PBS. Then, slides were incubated with MEM supplemented with 100 µg/mL rifampicin for 1 h and washed again with sterile PBS. To disrupt cells, 250 µL of 0.1% TritonX-100 was added and dilutions 1:1 to 1:5 were plated on LB agar to count CFUs [42].

### Biofilm formation assay

The ability to form biofilms was determined in a 96 wells plate. Bacteria were incubated in BHI (Brain–Heart Infusion) broth for 24 h and 48 h at 37 °C and biofilm formation was determined according with protocols previously reported [51, 52].

### Macrophage replication assay

J774 macrophages were grown in MEM supplemented with 10% FBS and incubated under a 5% CO_2_ atmosphere at 37 °C. Bacteria cultures were prepared inoculating 3 mL of LB broth with several *E. coli* colonies from LB agar plates. Macrophages were seeded into eight-well Millicel ^®^ EZ slide at 5 × 10^4^/mL and incubated overnight. The next day, macrophages were infected with *E. coli* strains at MOI (multiplicity of infection) of 100 and incubated for 3 h at 37 °C, 5% CO_2_. The medium was removed, and the cells were washed twice with sterile PBS and incubated with 100 µg/mL rifampicin in high glucose DMEM + 10% FBS for 1 h. The cells were washed twice with sterile PBS and lysed with 0.1% Triton X-100 for 10 min to release intracellular bacteria. Samples were serially diluted from 10^1^ to 10^4^ in PBS, plated on LB agar, and incubated at 37 °C overnight. Survival represents the product of invasion plus intracellular replication minus phagocytosis. Counts above 100 CFU indicate replication. All assays were done in triplicate in three independent trials [55].

### Gene sequencing

After PCR, genes of interest were purified with Zymo-Clean^®^ Gel DNA Recovery Kit (Zymo Research) and sequenced by Sanger methodology at Unidad de Sequenciación IBT-UNAM. Sequences were visualized and analyzed with FinchTV^®^ software and annealing packages from Clustall Omega.

### Statistical analysis

To determine significant differences between measures, two-way ANOVA analysis were performed with Bonferroni test, and with a 95% confidence interval with a P value < 0.01 using GraphPad^®^ from Prisma software package.

## Results

### *E. coli* ST131 O25:H4/H30-Rx virulence genes

The presence of 17 virulence genes was studied in our four bacteria strains, including *fimH*, *papA*, *iha*, *iucD*, *iutA*, *fliC*, *afa/draBC*, *afa* operon, *iroN*, *sat*, *ibeA*, *papGII cnfI*, *hlyA*, *papGIII*, *cdtB* and *neuC*-*KI.* A differential genetic presence was observed in two well defined virulence gene arrangements which do not correspond with the previously reported virotypes for *E. coli* ST131 [11]. The two new proposed patterns were virotype F found in C7226 and C7230 strains; and virotype G found in C7223 and C7225 strains (Tables 1 and 3).

**Table 3:** *E. coli* ST131 O25:H4/H30-Rx virotypes, according with the virulence genes content. Modified from Nicolas-Chanoine et al. [11]

Virotype	Virulence factors encoding genes										
	*afa/draBC*	*afa* operon	*iroN*	*sat*	*ibeA*	*papG*II	*cnfI*	*hlyA*	*papG*III	*cdtB*	*neuC*-K1
A	+	+	–	±	–	–	–	–	–	–	–
B	–	–	+	±	–	–	–	–	–	–	–
C	–	–	–	+	–	–	–	–	–	–	–
D	±	±	±	±	+	–	±	±	±	±	±
E	–	–	–	+	–	+	+	+	–	–	–
F^a^	–	–	+	–	–	+	+	–	–	–	–
G^a^	–	–	–	+	–	+	–	–	–	–	–

^a^new proposed virotype; +, positive PCR result; −, negative PCR result. *afa/draBC*, Afa/Dr adhesins; *afa* operon, FM955459; *iroN*, catecholate siderophore receptor; *sat*, secreted autotransporter toxin; *ibeA*, invasion of brain endothelium; *papG*II, allele II of *papG* gene; *cnf1*, cytotoxic necrotizing factor type 1; *hlyA*, alpha-hemolysin; *papG*III, allele III of *papG* gene; *cdtB*, cytolethal distending toxin; *neuC*-K1, K1 variant of group II capsule

The multiplex PCRs performed for *papA*, *papGII*, *papGIII*, *iha*, *satA, iucD*, *iutA*, *fliC*, *fimH* and for PAI-I and PAI-II of UPEC CFT073 and J96, were used to identify extra intestinal pathotypes. We amplified *papA*, *papGII*, *iha*, *satA*, *iucD* and *fimH* in all four *E. coli* ST131 strains; PAI-I and PAI-II of CFT073 for C7225, C7226 and C7230 (Table 1). Furthermore, PCRs for each diarrheagenic pathotype were performed and none of the four strains tested could be classified into these six pathotypes. These findings confirmed that all strains belong to extra intestinal pathotype.

### Relationship between the *E. coli* ST131 O25:H4/H30-Rx virotypes, the ESBL variants and its resistance genotype

As *E. coli* resistance traits has been linked with MGEs which also carry virulence determinants, we determined the association between resistance and virulence genes present among these four strains. Previously, Cortés-Cortés et al. [50] (Table 1) reported differential resistance patterns to β-lactamic and non β-lactamic antibiotics, phylogroup and *fimH* variant. Additionally, we performed PCR and sequencing of the *gyrA* and *parC* genes from all four healthy donors’ strains, and obtained the classical mutation (S80I and E84V for *parC* and S93L and E97N or S93I and E97V for *gyrA*) previously reported [53]. Because of the different resistance patterns in our four strains, we did not observe a relationship between resistance and virulence that suggested a co-occurrence of these traits in a MGE.

### Phenotyping of *E. coli* ST131 O25:H4/H30-Rx strains as AIEC pathotype

**Fig. 1:**
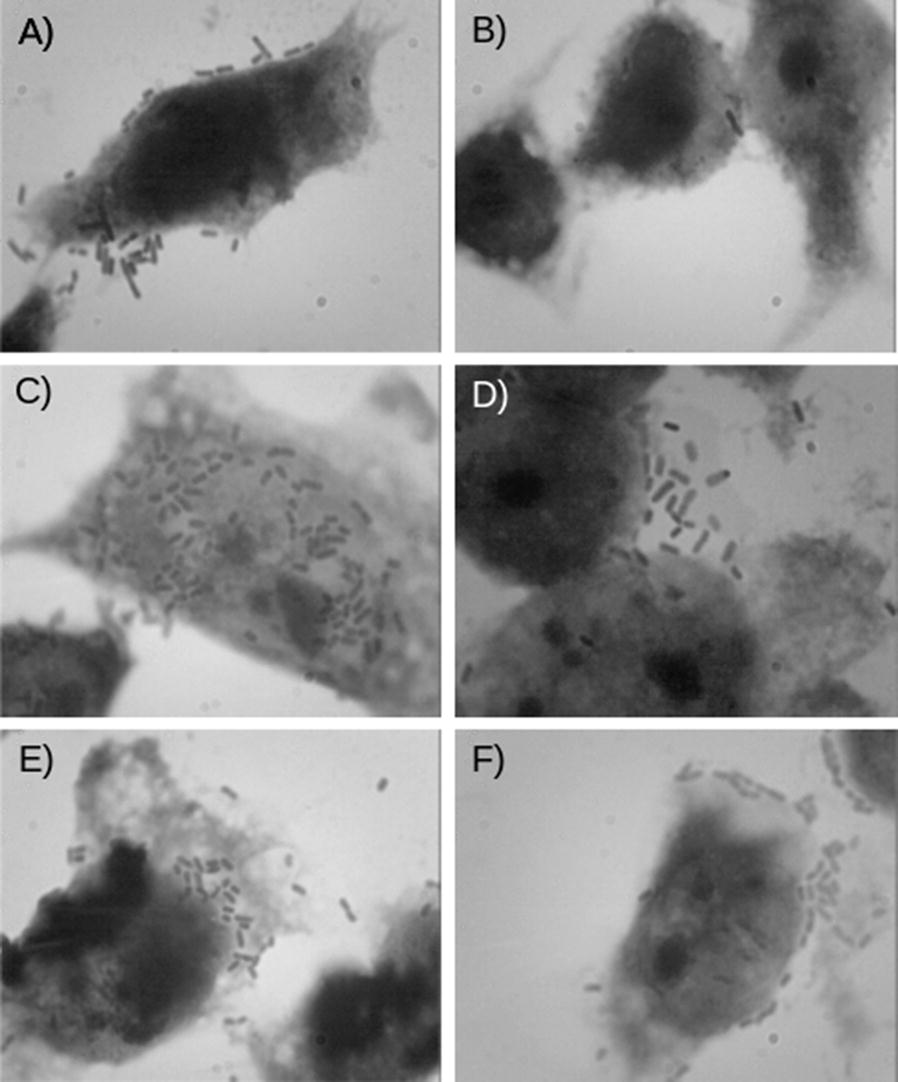
Adherence patterns observed in *E. coli* ST131 O25:H4/H30-Rx strains. **A** *E. coli* K-12, **B** UPEC CFT073, **C** C7223, **D** C7225, **E** C7226, **F** C7230. All strains were triple tested and stained with Giemsa

**Fig. 2:**
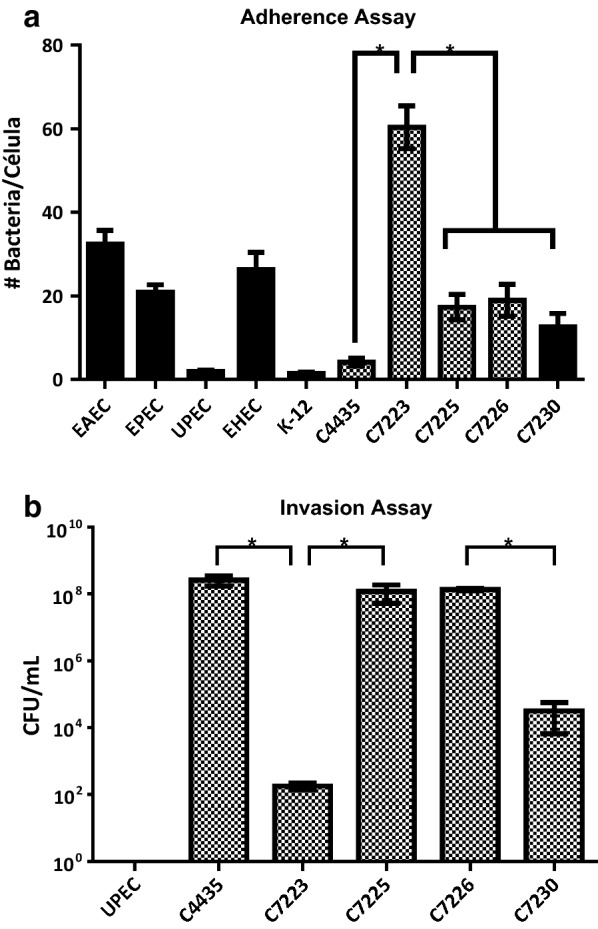
Adherence (**a**) and invasion (**b**) assays for the *E. coli* ST131 O25:H4/H30-Rx strains. All strains were triple tested. An * shows significant differences

**Fig. 3:**
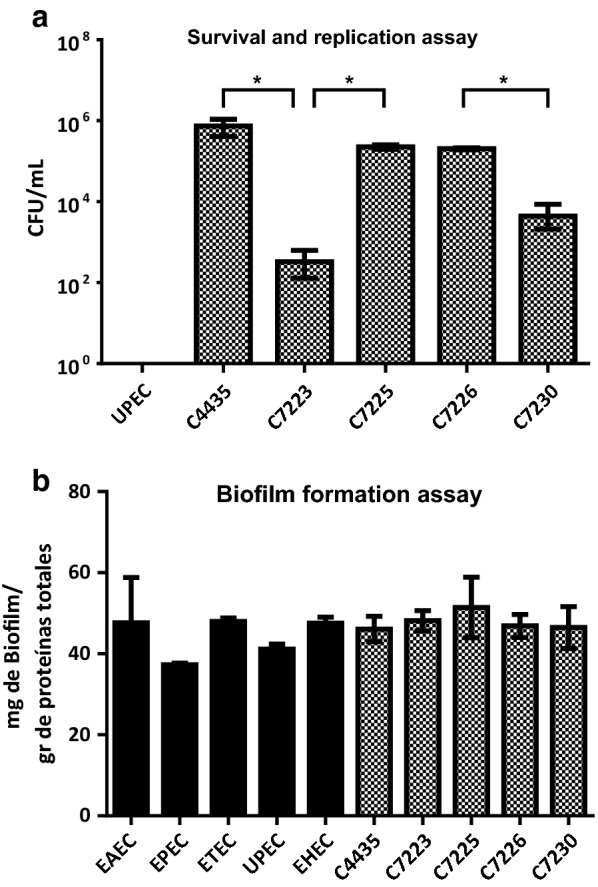
Survival within macrophages and Biofilm formation. *E. coli* ST131 O25:H4/H30-Rx within J774 macrophages (**a**), all strains were triple tested, and biofilm formation assay (**b**), all strains were tested eight times. An * shows significant differences

To evaluate these strains as potential members of AIEC pathotype, HeLa cells were infected with each strain and adhesion, invasion assays were performed (Fig. 1). C7223 strain showed the highest adherence levels (60.4 bacteria/cell) as compared to other adherent *E. coli* strains and thirty times higher than UPEC and K-12 strain. The other three strains showed less bacterial adherence per cell but more than the UPEC strain (Fig. 1). However, in invasion assays C7223 showed less invasiveness than the other three strains. Furthermore C7225 and C7226 showed 6 logs of difference with respect to the C7223 strain (Fig. 2b). Moreover, we tested the survival rate in macrophages. The survival rate in macrophages was also the highest in C7225 and C7226 strains (2.3 × 10^5^ and 2.0 × 10^5^ CFUs, respectively), followed by C7230 strain (4.4 × 10^3^ CFUs) and C7223 strain (3.3 × 10^2^ CFUs) (Fig. 3a and Table 4). These data confirm that these four strains of *E. coli* ST131 O25:H4/H30-Rx own similar phenotypic characteristics to the AIEC strains previously reported [42, 43].

**Table 4:** Characterization of *E. coli* ST131 O25:H4/H30-Rx strains with traits of the AIEC pathotype

Strain	Adherence rate^a^	Invasion rate^b^	Survival within macrophages^c^	Biofilm formation rate^d^
C7223	60.4 b/c	1.8 × 10^2^	3.3 × 10^2^	48.13
C7225	17.3 b/c	1.2 × 10^8^	2.3 × 10^5^	51.44
C7236	18.9 b/c	1.4 × 10^8^	2.0 × 10^5^	46.9
C7230	12.6 b/c	3.2 × 10^4^	4.4 × 10^3^	46.47

Determination of *E. coli* strains as belonging to the AIEC pathotype was performed using the following criteria: (1) the ability to adhere to HeLa cells with an adhesion index equal or superior to 1 bacteria per cell (b/c), (2) the ability of the bacteria to invade HeLa cells with an invasion index equal or superior to 0.1% of the original inoculum, (3) the ability to survive and to replicate within J774 macrophages

^a^Adherence rate is expressed as the mean of bacteria adhered to one HeLa cell

^b^Invasion is the mean CFUs after 1 h rifampicin treatment of infected HeLa cells

^c^Mean of intracellular bacteria at 3 h post infection relative to the number after 1 h rifampicin treatment, defined as 100% (MOI of 100)

^d^Biofilm formation rate is defined as milligrams per grams of total protein

### Biofilm formation of *E. coli* ST131 O25:H4/H30-Rx clones

Biofilm formation has been suggested as another feature of AIEC strains [54]. Here we tested if the four *E. coli* ST131 O25:H4/H30-Rx strains formed higher biofilms than non AIEC strains. Our results showed homogeneity in biofilm formation among the four strains (from 46 to 51 mg of biofilm/gr of total protein) which were higher but close to EPEC (37.3 mg), EHEC (47.58 mg) and ETEC (47.96). Interestingly, the four tested strains showed a lightly higher biofilm formation phenotype than UPEC (41.19 mg) and EAEC (47.64 mg) (Fig. 3b).

### *E. coli* ST131 O25:H4/H30-Rx strains harbors AIEC genetic determinants

We further examined *E. coli* ST131 O25:H4/H30-Rx strains for the presence of five genes of AIEC strains and widely characterized in the typical strains (NRG857C and LF82) [42, 49, 55, 56]. We amplified the *insA*, *insB*, *ibeA*, *intI*1 and *vat* genes, and found that all four strains tested were positive for the *insA*, *insB* and *intI*1 genes. None of the four strains carries *ibeA* and *vat* genes (Table 1). The non-pathogenic strain *E. coli* C600 was used as a negative control. These results indicate that the four *E. coli* ST131 O25:H4/H30-Rx strains we tested share genetic determinants similar with the AIEC strains previously characterized [42, 43, 49, 55, 56].

## Discussion

It is well documented that patients infected with ESBL-producing microorganisms have been erroneously cephalosporin treated or spent long time at hospital facilities, increasing the risk of develop selective pressure or acquiring resistant clones [57]. Interestingly healthy donors’ strains have shown resistance to β-lactamic, aminoglycosides and tetracycline. The high prevalence of CTX-M-15 and CTX-M-14 that can be carried in plasmid, indicates that could be acquired by clonal dissemination. This clones have been reported in healthy humans in Spain, Tunisia, China and The Netherlands but there is only two reports from Latin America [12, 50, 58–61].

There are reports indicating that *E. coli* ST131-B2 are multi-drug resistant, harboring plasmids carrying on *bla*_*CTX*-*M*-*15*_ gene from clinical samples, healthy humans and soil isolates [8, 12, 62, 63]. The four strains tested belong to O25:H4 serotype and *fimH* 30 variant, as they showed β-lactamase and quinolone resistance belonging to a subclone ST131-B2-O25:H4/H30-Rx, which is recognized for increasing rates of morbidity, mortality and costs in the clinical area and community [11]. Strains belonging to O25:H4 serotype are usually related with high virulence rates, and here we identified two new virotypes with different genetic arrangements, suggesting an endemic distribution of virulence genes probably acquired by MGEs. We also detected genes related to ExPEC strains used as PAI markers, which would explain the high virulence reported for ST131 strains [8, 62]. Nevertheless, it has been proposed that acquisition of virulence gene determinants such as *afa/draBC*, *afa* operon, *iroN*, *sat*, *ibeA*, *papGII*, *cnf1*, *hlyA*, *papGIII*, *cdtB* or *neuC*-*K1* in ST131 strains was prior to the development of resistance to fluoroquinolones, causing this clone to emerge steadily, first acquiring genes associated with its ability to cause infections in humans and then endowing itself with an arsenal of antimicrobial resistance that has trigger its massive expansion worldwide [11, 64]. When we looked for specific pathotype genes, PAI-I and II from UPEC CFT073 were identified in three of the four strains (C7225, C7226, C7230), additionally the fact that strain C7223 had a different ST could explain the variability of housekeeping genes tested when determining sequence typing; however, despite of the belonging to specific virotypes, we were not able to correlate resistance with virulence pattern.

AIEC recently described, are not associated with diarrhea, and instead they are thought to contribute to the development of chronic inflammatory bowel diseases such as in the case of Crohn’s disease and ulcerative colitis. AIEC strains can be identified by their ability to adhere to and invade epithelial cells and to replicate within macrophages and for harboring genetic determinants as *insA*, *insB*, *intI*1, *ibeA* and *vat* [42, 49, 55, 56, 65]. Analysis of whole genome sequences of several AIEC isolates had shown that the AIEC phenotype may not be due to one or more specific virulence determinants, suggesting that the distinctive phenotype of these bacteria may result from metabolic processes that enhances growth in tissues affected by Crohn’s disease. Thus, although AIEC are recovered more commonly from patients with Crohn’s disease than from healthy people, we identified four strains from healthy donors that did not referred any symptoms at the time of collecting the sample, nevertheless, it is common for the Mexican population to have food consumption habits with a high amount of irritants such as alcohol and spice which could predispose to inflammatory conditions, that together with this type of bacteria complicate the development of the illness supporting dysbiosis events that have been recently proposed [48].

In this study, we determined that the four strains survived differentially within macrophages. This trait has been related to *ompA* and *ompC* expression [66], so it will be interesting, in future experiments, to look for the expression of these genes in the four strains under infection conditions. Recently, have been reported the rise of strains that show increased catalytic efficiencies toward extended-spectrum cephalosporin known as ESAC (extended-spectrum AmpC) producing strains [67], evidence suggests the loss of OmpC and OmpF porins but also mutations at *ampC* promoter level; interestingly, three of the four strains tested showed cefepime resistance what makes them candidates to ESAC-producing *E. coli*, then it will be interesting too, in future experiments, to determine the sequence changes and to evaluate it with mutagenesis-complementation assays. Furthermore, biofilms are communities of microbes attached to surfaces and have a few distinct characteristics; they are typically surrounded by an extracellular matrix that provides structure and protection to the community; bacteria growing in a biofilm also have a characteristic architecture generally comprised of macrocolonies containing thousands of cells surrounded by fluid-filled channels; biofilm-grown bacteria are also notorious for their resistance to a range of antimicrobial agents including clinically relevant antibiotics [54]. Previous work showed a higher to form biofilm capacity amongst AIEC than non-AIEC strains, suggesting this feature as an important determinant involved into AIEC pathogenesis [54]. Our four strains showed high rates of fluoroquinolone resistance [50] only detecting punctual mutations in *gyrA* and *parC* genes; it can be explained by its biofilm formation capability, however, the biofilm formation rates of the four tested strains were similar to those showed by the control strains, so we cannot consider it as an indicative characteristic of AIEC pathotype. It would be interesting to extend the research of this pathotype to other sources (such as urine), since these strains harbor pathogenic determinants that could confer them the ability to invade/colonize which could lead to a clinical picture.

To our knowledge, this report represents the first characterization of AIEC in Mexico and the first time these strains are isolated from healthy donors; moreover, it is the first detection of an AIEC strain related to ST131 clone. This, together with the findings of new virotypes, highlights the importance of these strains as reservoirs or carriers of MDR and highly infective strains that could be transmitted to vulnerable population.
